# Anti-Corrosion Performance of Migratory Corrosion Inhibitors on Reinforced Concrete Exposed to Varying Degrees of Chloride Erosion

**DOI:** 10.3390/ma15155138

**Published:** 2022-07-24

**Authors:** Congtao Sun, Ming Sun, Junde Liu, Zhenping Dong, Liang Fan, Jizhou Duan

**Affiliations:** 1 Key Laboratory of Marine Environmental Corrosion and Bio-Fouling, Institute of Oceanology, Chinese Academy of Sciences, Qingdao 266071, China; sunmingfx@126.com (M.S.); fanl@qdio.ac.cn (L.F.); duanjz@qdio.ac.cn (J.D.); 2 Open Studio for Marine Corrosion and Protection, Pilot National Laboratory for Marine Science and Technology (Qingdao), Qingdao 266237, China; 3 School of Civil Engineering, Xi’an University of Architecture and Technology, Xi’an 710055, China; mw_409250288@163.com

**Keywords:** anti-corrosion performance, MCIs, reinforced concrete, chloride erosion, steel bar

## Abstract

This study investigated the anti-corrosion performance of commercial amino alcohol migratory corrosion inhibitors (MCIs) on concrete that underwent varying degrees of chloride erosion. Electrochemical impedance spectroscopy (EIS), potentiodynamic polarization (PD), scanning electron microscopy, and energy dispersive spectroscopy (SEM-EDS) analyses were performed to study the anti-corrosion performance and mechanism of the MCIs on the steel bars. The results indicated that the corrosion resistance of the steel bars in concrete was significantly improved by coating with the MCIs, and the earlier the specimens were coated with the MCIs, the higher the anti-corrosion efficiency. The anti-corrosion efficiency was 55.35% when the MCIs coating was applied before chloride erosion; however, the anti-corrosion efficiency decreased to 3.40% when the MCIs coating was applied after the ninth drying–wetting cycle. The improvement in corrosion resistance of the steel bar in concrete coated with MCIs was due to the protective MCIs-molecule film that formed on the steel bar surfaces, and the oxidative dissolution of iron at the anode was effectively inhibited by the MCIs coating.

## 1. Introduction

Steel-reinforced concrete is widely used in the construction industry, owing to its applicability and durability. A protective passive film forms on the steel rebar surfaces due to the alkaline concrete pore solution [1,2]. However, in marine environments or due to the application of de-icing salts, the passive film is degraded if chloride concentrations exceed the threshold value; this would lead to the corrosion of the steel bar and reduced service life of the concrete structures [3,4,5]. To mitigate chloride-induced corrosion, migratory corrosion inhibitors (MCIs) can be used; this is one of the most effective and recognized methods [6,7].

Compared to other approaches for mitigating chloride-induced corrosion, such as the use of high-performance concrete, sacrificial anodes, and electrochemical chloride extraction [8,9,10], the MCIs can be applied to the concrete surface as well as an admixture, are highly economical, and are easy to use [11]. Moreover, the MCIs can be applied to both relatively young and hardened concrete structures [6]. The MCIs molecules can reach the steel bar surface through capillary infiltration and vapor diffusion, forming a compact layer to protect the steel bar from corrosion [2,12]. In addition, the pore-blocking effect on the concrete surface via MCIs coating can not only prevent the evaporation of the MCIs molecules from the concrete, but also reduce the penetration of aggressive ions [13,14].

Many researchers have conducted significant investigations on the anti-corrosion performance and mechanism of MCIs on steel bars under chloride erosion. The frequently-used MCIs are amine salts, amino-alcohol, and organo-functional-based inhibitors [12,15]. Studies have typically adopted two experiment methods, where the steel bars are immersed in a simulated concrete pore solution or embedded in cementitious materials. Zheng et al. [2] used saturated Ca(OH)_2_ solutions to simulate a concrete pore solution, and they found that the MCIs protected the steel bar both before and after chloride erosion. Tiwari et al. [16] investigated the corrosion inhibition efficiency of the inhibitors by immersing steel bars in a carbonated pore solution with chloride ions and found that the inhibitor was efficient in retarding the corrosion of the steel bar in a combined chloride and carbonated environment. For steel bars embedded in mortars or concrete, Malik et al. [17] studied the performance of two types of amino alcohol-MCIs in the protection of steel in a marine environment, and they observed a significant decrease of corrosion rates of and very little or no corrosion in MCIs-treated samples. Goyal et al. [12] found that the organo-functional silanes MCIs also had higher corrosion resistant and barrier properties due to their water repellent nature. Moreover, the migration efficiency of the MCIs was closely related to the concrete thickness, w/c, and strength of concrete [18,19]. In addition, the green corrosion inhibitor containing green compounds and natural substances typically extracted from plants was reportedly effective in reducing the risk of chloride-induced steel corrosion [20,21]. Compared to applying the MCIs on a concrete surface, there are some drawbacks of using them as an admixture. The function groups in MCIs, such as amino-alcohol, carboxylic acids, and hydroxyl groups, show negative effects on fresh concrete by chemically interacting with hydrated and unhydrated phases of cement [16,22]. However, these negative effects can be avoided by applying MCIs on the hardened concrete surface [23]. More importantly, the corrosion of the steel bars could occur during their service life, and the corrosion degree may vary depending on the degree of chloride erosion. Therefore, the inhibitive action of MCIs on concrete that has undergone varying degrees of chloride erosion requires further exploration.

In this research, the different drying–wetting cycles were used to simulate the different degrees of chloride erosion, and the anti-corrosion performance of the MCIs on concrete that was exposed to varying degrees of chloride erosion was investigated. The electrochemical performance was then investigated as a function of MCIs’ application after the concrete underwent varying degrees of chloride erosion, and electrochemical impedance spectroscopy (EIS) and potentiodynamic polarization (PD) studies were used in this study. Additionally, the microscopic morphology and elemental content of the steel bars were analyzed by scanning electron microscopy with energy dispersive spectroscopy (SEM-EDS).

## 2. Materials and Methods

### 2.1. Materials and Specimen Preparation

Ribbed Q235 (Boyuan, Qingdao, China) steel rebars, 8 mm in diameter and 30 cm in length, were used to prepare the steel-reinforced concrete. Before casting, the steel rebars were polished with sandpaper and cleaned with ethanol, and the copper wires were soldered to one end of the sections. The middle 200 mm section of the steel rebars was used as the erosion area, while the remainder was first coated with epoxy resin and then wrapped with insulating tape. Ordinary Portland cement (OPC, grade 42.5, China united cement, Rizhao, China) and type I fly ash were used in this study, and their main chemical compositions measured by an X-ray fluorescence spectrometer (Bruker, Karlsruhe, Germany) are listed in Table 1. The coarse aggregates consisted of crushed stone with a particle size of 5–20 mm, and river sand was used as the fine aggregate, with a fineness modulus of 2.7. Tap water was used for mixing the concrete, and a polycarboxylic acid high-performance water reducer was used to adjust the fluidity of the mixtures. The mix proportion was designed according to the JGJ-2011 standard of China (specification for mix proportion design of ordinary concrete) and is listed in Table 2. The fresh concrete was poured into plastic molds after the completion of mixing. The specimens were prepared with a size of 100 × 100 × 300 mm to determine the electrochemical information and micromorphology of the steel bar, and two thin plastic plates were used to fix the steel rebar with a protective depth of 15 mm. Furthermore, specimens were prepared with a size of 150 × 150 × 150 mm to determine compressive strength. After 24 h of curing at room temperature, all concrete specimens were removed from the molds and placed in a curing room at 20 ± 3 °C and relative humidity (RH) of ≥95% for 27 d. After standard curing, the average concrete compressive strength was 48.2 MPa, measured with a compressive-testing machine. Commercial amino alcohol-MCIs ( Sika, Shanghai, China) were used in this study, and the dosage of MCIs was 500 g/m^2^, according to the manufacturer’s recommendations.

### 2.2. Chloride Erosion Test

Before chloride erosion, the five specimen surfaces were sealed with epoxy resin, leaving only one polished surface as the exposed surface. The chloride erosion test was conducted according to the Chinese standard GB/T 33083-2017 (technical specification for application of reinforced concrete anti-corrosion inhibitor). Different drying–wetting cycle times were used to simulate the varying degrees of chloride erosion. The first drying–wetting period was 64 h, where the specimens were immersed in a sodium chloride solution with a concentration of 3.5% with a pH value of 12.3 for 16 h. Then, the specimens were removed from the solution and placed in an oven at a temperature of 60 °C for 48 h. Specimens A1, A2, A3, A4, and A5 were coated with the MCIs after the first, third, fifth, seventh, and ninth drying–wetting cycles, respectively. Specimen B was coated with the MCIs before the drying–wetting cycles; A0 was used as the control, but it was not coated with MCIs. Three parallel samples were prepared for each condition.

### 2.3. Electrochemical Tests

Electrochemistry tests were performed using a three-electrode electrochemical cell. The steel bar in the concrete was the working electrode; the saturated calomel electrode was used as the reference electrode, and the graphite electrode served as the counter electrode. Prior to the electrochemistry tests, the specimens were immersed in sodium chloride solution for 30 min to keep them moist, and the sodium chloride solution was used as the electrolyte, and the wires were polished bright to ensure good conductivity. In addition, the variations in open circuit potential (OCP) did not exceed more than 2 mV within 5 min before the electrochemistry tests. For the EIS tests, the sinusoidal excitation voltage amplitude was 10 mV, and the frequency range was from 0.01~10^5^ Hz. The PD test was performed at a scan rate of 0.166 mV/s from −250 mv vs. OCP to +250 mv vs. OCP. All of the electrochemistry tests were performed using a PARSTAT 4000 electrochemical workstation (AMETEK, Princeton, NJ, USA). 

### 2.4. SEM-EDS Tests

After 11 drying–wetting cycles, all of the concrete specimens were split, the steel rebars were removed, and 10 mm was cut out. The morphological characterization and elemental analysis of the steels were performed by SEM-EDS in this study, using a ZEISS-EVO18 scanning electron microscope (ZEISS, Oberkochen, Germany).

## 3. Results and Discussion

### 3.1. EIS Studies

**Figure 1:**
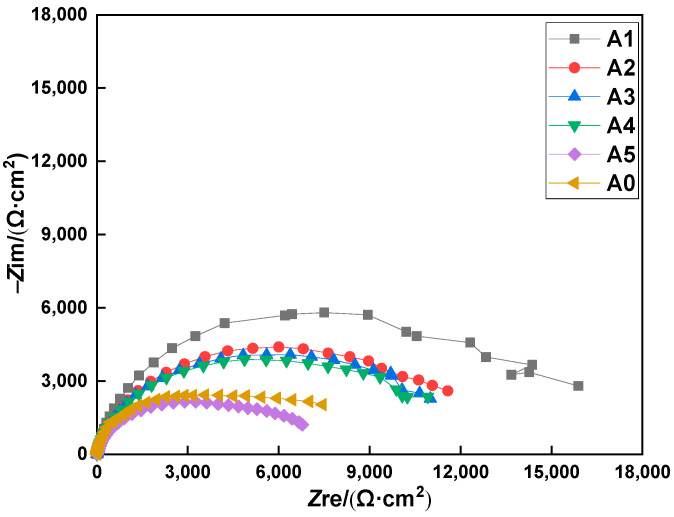
Nyquist plots of the MCIs coated specimens after different degrees of chloride erosion.

For group A, the anti-corrosion performance evaluation of the MCIs was carried out by the EIS studies after 11 drying–wetting cycles. The Nyquist plots of the specimens coated with the MCIs after varying degrees of chloride erosion are shown in Figure 1. Figure 1 also shows that the impedance arc radius of sample A1 was significantly greater than the others, indicating that the inhibitor exhibited good anti-corrosion performance with the MCIs coating after the first drying–wetting cycle. Furthermore, the impedance arc radii of A2, A3, and A4 were greater than A0, which indicated that the corrosion of the steel rebar was also inhibited after the third, fifth, and seventh drying–wetting cycles when it was coated with the MCIs. Moreover, the impedance arc radius gradually decreased with delayed MCIs coating time, and the impedance arc radii of samples A5 and A0 were not significantly different. This indicated that the anti-corrosion effect of MCIs gradually weakened with delayed coating time, and the inhibitor did have an anti-corrosion effect with the MCIs coating after the ninth drying–wetting cycle.

**Figure 2:**
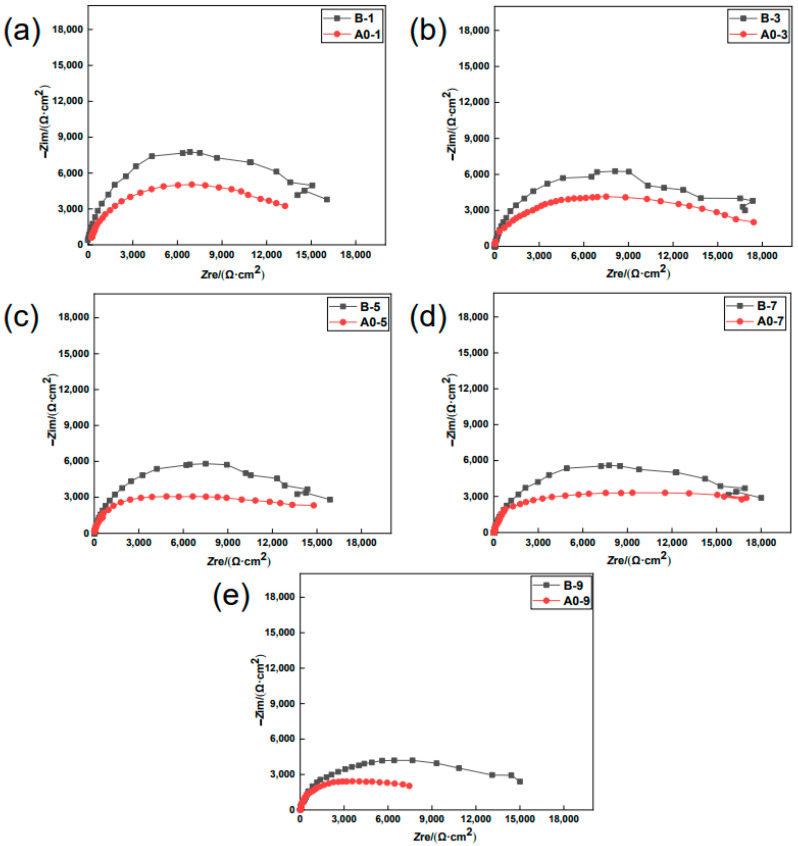
Nyquist plot of B versus A0 at different degrees of chloride erosion (where 1, 3, 5, 7, and 9 represent the specimens coated with MCIs after the 1st (**a**), 3rd (**b**), 5th (**c**), 7th (**d**), and 9th (**e**) drying-wetting cycles, respectively).

**Figure 3:**
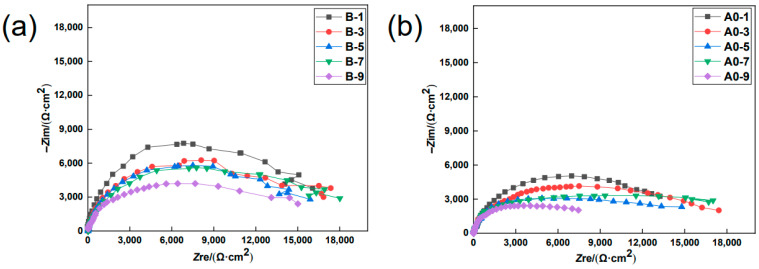
Nyquist plot of B (**a**) and A0 (**b**) with an increasing number of drying–wetting cycles.

To meticulously investigate the evolution of anti-corrosion performance by the MCIs coating during the chloride erosion process, specimens B and A0 were assessed by EIS studies after a different number of drying–wetting cycles. Figure 2 shows the Nyquist plot of B versus A0 at different degrees of chloride erosion. As shown in Figure 2, the impedance arc radius of B was significantly greater than A0 at the same drying–wetting cycle. This indicated that the MCIs coating before chloride erosion had a good anti-corrosion effect throughout the entire chloride erosion process. Moreover, the impedance arc radii of B and A0 gradually decreased during the chloride erosion process, as shown in Figure 3, which indicated that the degree of steel corrosion gradually increased with chloride erosion.

**Figure 4:**
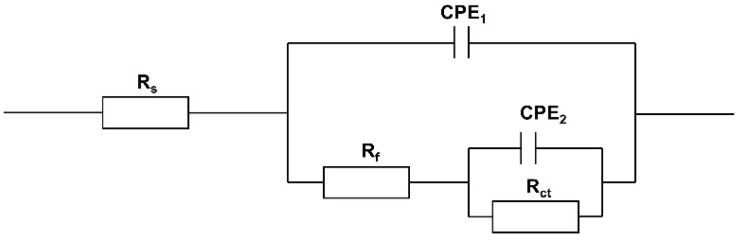
Equivalent circuit model used to fit EIS results.

To quantitatively analyze the anti-corrosion performance of MCIs on the reinforced concrete that underwent varying degrees of chloride erosion, the equivalent circuit model shown in Figure 4 was used to fit the Nyquist plots [24,25], and the fitted results are listed in Table 3. In the equivalent circuit model in Figure 4, *R*_s_ denotes the resistance of the sodium chloride solution during chloride erosion, *R*_f_ denotes the resistance of the protective MCIs-molecule film on the steel bar surface, and *R*_ct_ denotes the charge transfer resistance. The *CPE*_1_ denotes the constant phase angle element constituted by the film capacitance *C*_f_ and dispersion index *n*_1_, and the *CPE*_2_ was constituted by the double layer capacitance, *C*_dl_, and dispersion index, *n*_2_. The anti-corrosion efficiency, *η*, was calculated by Equation (1):

(1) $$\eta =\frac{R_{\mathrm{ct}}-R_{\mathrm{ct}}^{0}}{R_{\mathrm{ct}}}\times 100\%$$

where *R*_ct_ and *R*^0^_ct_ refer to the charge transfer resistance of the MCIs coated and uncoated steel bar in the concrete, respectively.

As shown in Table 3, the *R*_ct_ (kΩ/cm^2^) values of B, A1, A2, A3, A4, and A5 were 7.01, 4.54, 4.37, 4.29, 4.00, and 3.24, and the *η* values were 55.39%, 31.06%, 28.38%, 27.04%, 21.75%, and 3.40%, respectively. These results indicated that the earlier the specimen was coated with the MCIs, the higher the charge transfer resistance and the better the anti-corrosion efficiency. Coating with MCIs before chloride erosion showed the highest anti-corrosion efficiency; however, the anti-corrosion efficiency was reduced when the MCIs-coated specimens suffered some degree of chloride erosion. Specifically, coating with the MCIs after the ninth drying–wetting cycle had little effect on anti-corrosion.

In general, the above results indicated that the corrosion resistance of the steel bar was significantly improved by coating with the MCIs at low chloride erosion levels; however, the improvement gradually decreased with an increasing degree of chloride erosion. In particular, the corrosion resistance of the steel bar did not improve after coating with MCIs at higher levels of chloride erosion. Thus, a critical level of chloride erosion could exist under which the corrosion resistance of the steel bar would be improved by coating with MCIs, and above this level, the corrosion resistance could not be improved. Due to the interactions between the MCIs molecules and the steel surface, a protective MCIs molecule film would form on the steel bar surface, preventing chloride erosion [2,12]. However, due to the reactions among the salt-forming components in MCIs and calcium hydroxide, gel formation could block the concrete pores, preventing the evaporation of the MCIs, as well as chloride penetration [13,14]. Therefore, compared to A0, the corrosion resistance of B, A1, A2, A3, and A4 significantly improved by coating with the MCIs. However, there was still competitive adsorption between the MCIs molecules and the chloride ions on the steel bar surface, which not only reduced the already present MCIs-molecule film stability, but also decreased the compaction of the MCIs-molecule film during the formation process [2,6]. Therefore, the degree of steel corrosion increased gradually with chloride erosion, and the anti-corrosion effect of the MCIs gradually weakened with delayed coating time.

### 3.2. PD Studies

**Figure 5:**
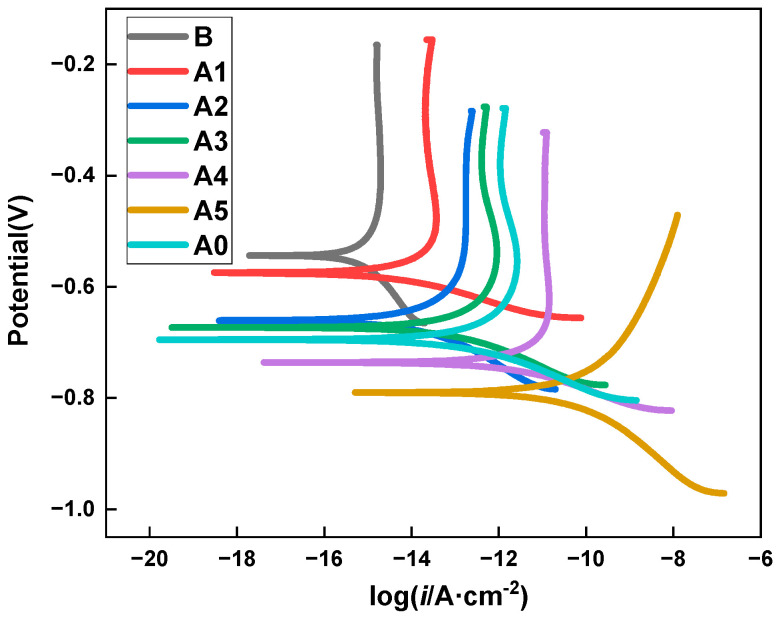
Potentiodynamic polarization curves of specimens coated with MCIs after different degrees of chloride erosion.

The potentiodynamic polarization curves of the specimens after 11 drying–wetting cycles are displayed in Figure 5. Compared to the specimen without MCIs, a wide passive range appeared in the specimens coated with MCIs, as shown in Figure 5. This was mainly attributed to the compact and uniform passive film formed on the steel in the specimens coated with MCIs, and the inhibitor molecule absorbed on steel surface led to the formation of passive film within a short time [26]. In addition, the polarization curves moved to a more positive direction after coating with the MCIs, and the earlier the specimens were coated with the MCIs, the higher the corrosion potential. These results indicated that the corrosion resistance of the specimens was effectively improved by coating with the MCIs, and the earlier the specimen was coated with the MCIs, the higher the corrosion resistance. It was also observed that compared to the cathodic reactions, the corrosion current densities for the anodic reactions decreased significantly by coating with the MCIs, indicating that the oxidative dissolution of iron at the anode was inhibited by the protective MCIs-molecule film, which formed on the steel bar surface. All of these results were generally in good agreement with the above analysis based on the EIS studies.

### 3.3. SEM-EDS Analysis

**Figure 6:**
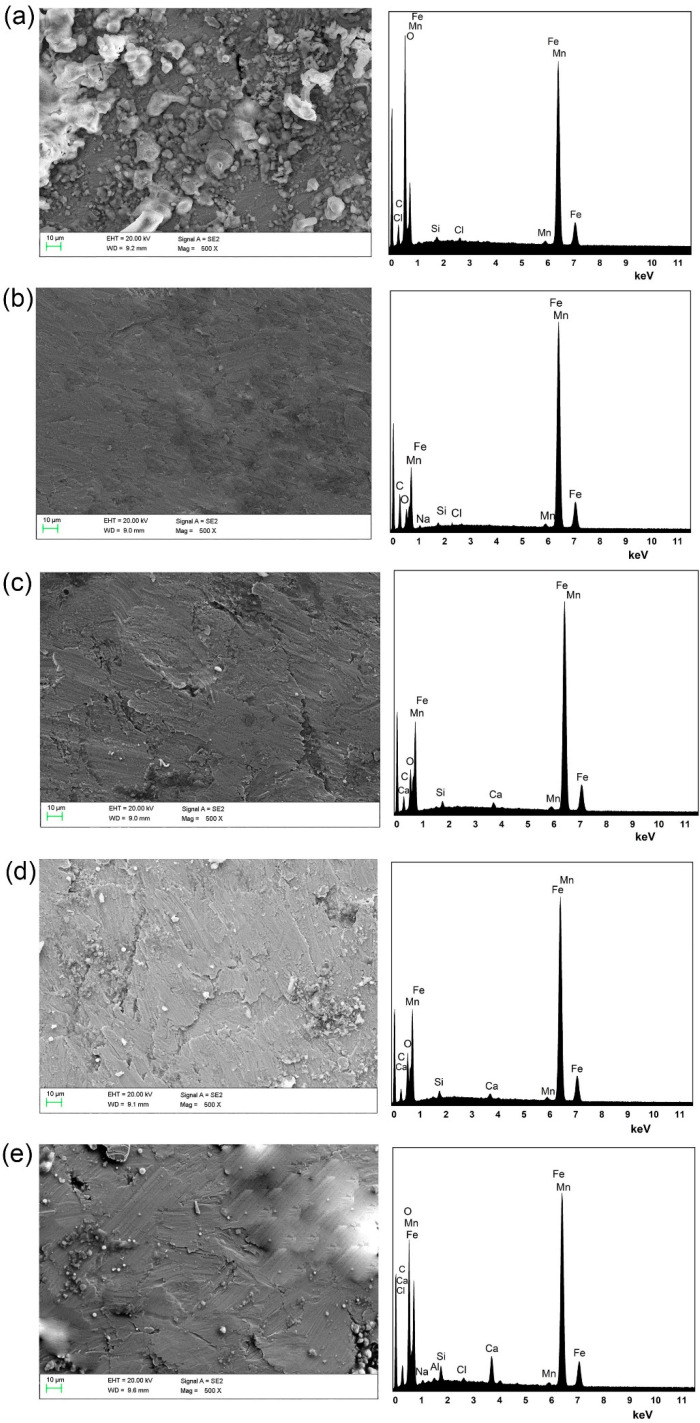

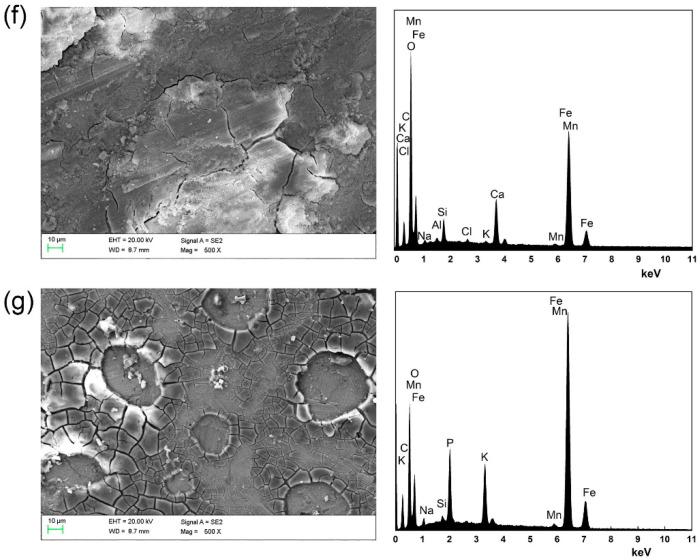
SEM and EDS analysis of the specimens coated with MCIs after varying degrees of chloride erosion; (**a**) A0-steel bar, (**b**) B-steel bar, (**c**) A1-steel bar, (**d**) A2-steel bar, (**e**) A3-steel bar, (**f**) A4-steel bar, (**g**) A5-steel bar.

The images for SEM-EDS analysis of the steel bars are shown in Figure 6, and the elemental contents on the steel bar surfaces from EDS analysis are shown in Table 4. As shown in Figure 6a, a large number of corrosion products piled up on the steel bar surface, and a mud-crack morphology of the corrosion products was observed, suggesting the formation of a corrosion product layer. According to the EDS results, the elemental Fe content values on the steel bar surfaces in specimens A0 was 34.65%. These results indicate that a severe corrosion reaction occurred on the steel bar in the specimens without MCIs coating. By contrast, as shown in Figure 6b,c, very few corrosion products were observed on the steel bar surface. In addition, the elemental Fe content values on the steel bar surfaces in specimens B and A1 were 73.52% and 68.97%, which were far higher than in A0. This indicated that the oxidative dissolution of iron ions was effectively inhibited after coating with the MCIs. Furthermore, the elemental C content values on the steel bar surfaces in B and A1 were also higher than in A0, indicating that a large number of organic substances were present on the steel bar surfaces in B and A1. This was attributed to the protective MCIs-molecule film that formed on the steel bar surfaces, which can hinder the corrosion of chloride ions. Notably, there was 0.32% Cl content on the surfaces of the steel bars in A0, and no Cl content was observed in B and A1. This also proved that the MCIs-molecule film was present on the steel bar surfaces, which effectively hindered the absorption of chloride ions on the steel bars.

As shown in Figure 6d,e, corrosion products and pitting gradually appeared on the steel bar surfaces when the MCIs coating time was delayed. Furthermore, a corrosion product layer with small rips was observed on the steel bars in specimens A4. Moreover, a corrosion product layer with large rips and pits was observed on the steel bars in specimens A5, which could be attributed to the abscission of loose corrosion products. These results indicated that the corrosion inhibition effect of MCIs gradually decreased with delayed MCIs coating time. These findings were consistent with the electrochemical results.

## 4. Conclusions

The anti-corrosion performance and mechanism of the MCIs on reinforced concrete with different degrees of chloride erosion were investigated via electrochemical measurements and SEM-EDS studies. According to the above results and discussion, the following conclusions can be drawn.

1. The corrosion resistance of the steel bars in the concrete significantly improved after coating with the MCIs. The protective capacity performed well not only after coating with the MCIs before chloride erosion but also after the concrete underwent varying degrees of chloride erosion.
2. The EIS results show that the earlier the specimen was coated with the MCIs, the higher the obtained anti-corrosion efficiency became. When the specimens were coated with the MCIs before chloride erosion, the charge transfer resistance value of the steel bar and the anti-corrosion efficiency were 7.01 kΩ/cm^2^ and 55.39%, respectively; however, the charge transfer resistance values and the anti-corrosion efficiency decreased to 3.24 kΩ/cm^2^ and 3.40% when the MCIs coating was applied after the ninth drying–wetting cycle.
3. The PD results revealed that the oxidative dissolution of iron by the anode under chloride erosion was effectively inhibited due to a protective MCIs-molecule film that formed on the surface of the steel bar.
4. The SEM-EDS results showed that there were very few corrosion products on the steel bar surface in specimens coated with the MCIs before chloride erosion, while the corrosion product gradually accumulated on the steel bar surface with the delayed MCIs coating time. The element Fe content values were 73.52% on the steel bar surfaces in specimens coated with the MCIs before chloride erosion, which gradually decreased with the delayed MCIs coating time.

## Figures and Tables

**Table 1:** Chemical compositions of the raw materials (wt%).

Material	SiO_2_	Al_2_O_3_	Fe_2_O_3_	CaO	MgO	SO_3_	K_2_O	TiO_2_	P_2_O_5_	Total
OPC	19.90	4.14	2.82	63.27	1.60	4.49	0.73	-	-	98.55
FA	43.90	43.80	2.71	3.31	0.54	0.75	0.82	1.63	0.74	98.2

**Table 2:** Mix proportion (kg/m^3^).

Cement	Fly Ash	Water	Sand	Gravel	Water Reducer
290	65	160	750	1150	5.4

**Table 3:** Fitting values of the Nyquist plots after 11 drying–wetting cycles.

Name	*R*_s_ (Ω·cm^−2^)	*C*_f_ (μF·cm^−2^)	*n*_1_	*R*_f_ (kΩ·cm^−2^)	*C*_dl_ (μF·cm^−2^)	*n*_2_	*R*_ct_ (kΩ·cm^−2^)	*η* (%)
A0	11.23	4.281	0.50	2.82	6.988	0.48	3.13	--
B	12.24	4.719	0.35	5.46	1.284	0.59	7.01	55.35
A1	11.22	5.482	0.35	4.08	2.337	0.55	4.54	31.06
A2	11.01	5.384	0.64	3.49	6.539	0.46	4.37	28.38
A3	12.02	5.100	0.49	3.40	6.813	0.48	4.29	27.04
A4	12.22	4.591	0.31	2.48	3.945	0.70	4.00	21.75
A5	11.02	4.450	0.34	9.94	1.278	0.60	3.24	3.40

**Table 4:** Elemental content on the steel bar surfaces, as obtained by EDS (at. %).

Elements	Fe	O	C	Cl	Mn	Ca	Others
A0	34.65	61.51	2.52	0.32	0.57	0.00	0.43
B	73.52	12.78	9.52	0.00	1.03	0.00	3.15
A1	68.97	24.20	3.50	0.00	1.00	0.93	1.40
A2	51.14	40.90	2.32	0.00	0.67	1.45	3.52
A3	37.44	53.19	2.64	0.35	0.49	2.63	3.26
A4	20.39	69.16	2.44	0.25	0.27	3.88	3.61
A5	35.31	46.49	3.68	6.88	0.46	0.00	7.18

## Data Availability

The data that support the findings of this study are available from the corresponding author upon reasonable request.
